# The Use of AMH to Assess Ovarian Toxicity in Adolescents and Young Adults After Cancer Treatment

**DOI:** 10.1210/clinem/dgaa277

**Published:** 2020-05-18

**Authors:** Richard A Anderson, W Hamish B Wallace

**Affiliations:** 1 MRC Centre for Reproductive Health, Queen’s Medical Research Institute, University of Edinburgh, Edinburgh, Scotland; 2 Department of Oncology and Haematology, The Royal Hospital for Sick Children, Edinburgh, Scotland

The field of pediatric oncology has assimilated the overlapping concepts of survivorship and late effects, recognizing that a key objective of treatment is to increase the number of young people surviving while minimizing long-term disadvantage and ill health (1). This concept is evolving in the care of young adults with cancer, partly spurred on by the growing awareness of patients and practitioners of the potential of loss of fertility and the potential to preserve it (2). Implicit in this is the need for an accurate assessment of the female reproductive function that ideally would estimate both immediate and long-term fertility, and the remaining reproductive lifespan. This also has relevance to wider aspects of women’s health, most clearly established for bone health, but likely also to have implications for cardiovascular and cognitive function, and indeed overall lifespan (3).

The dynamic nature of ovarian function means that there are large variations in conventional markers of ovarian activity, notably estradiol and follicle stimulating hormone. These largely reflect the latest stages of follicle growth in relation to ovulation, and it has really been the advent of the measurement of anti-Müllerian hormone (AMH) that has given us the opportunity to explore the activity of the ovary in terms of its smaller follicles (4). Measurement of AMH has become routine in assisted reproduction as a predictor of the ovarian response to stimulation, but its potential role in diagnosing—or indeed predicting—menopause in healthy women and premature ovarian insufficiency (POI) in patients diagnosed and treated for cancer have also been recognized. It is also clear that AMH is of no value as a predictor of short-term fertility (5). In women with cancer, AMH falls markedly during chemotherapy, with variable recovery thereafter depending on the degree of gonadotoxicity of the treatment administered (6). This has been demonstrated in prepubertal girls, in young adults, and in older premenopausal women, but generally in relatively small studies, particularly when prospective and mostly with less than a 5-year follow-up. The question of the longer-term function of the chemotherapy-exposed ovary therefore remains very uncertain, and it is this that Su and colleagues have investigated in a paper in the current edition of the Journal of Clinical Endocrinology and Metabolism (7). The study recruited women who had been diagnosed with cancer from the ages of 18 to 39 who were on the registries of 2 US states or were known to participating research centers. A total of 763 women participated and provided blood samples in the form of dried blood spots up to 15 years from diagnosis, with individual women contributing up to 4 samples (although approximately a third contributed only 1); AMH concentration was calculated as a serum-equivalent value. Importantly, the diagnosis and details of treatment were ascertained by physicians rather than self-reported. The treatment exposure was stratified into 3 groups defined as low, moderate, or high gonadotoxicity. The low group contains treatments that essentially were not gonadotoxic, including surgery only (excluding hysterectomy and/or oophorectomy), endocrine therapy only, radioiodine treatment, and cervical trachelectomy. The high gonadotoxic treatments included any exposure to pelvic radiation, stem cell or bone marrow transplant, or cyclophosphamide equivalent dose (CED) of >7 grams/m^2^. All other patients were stratified into the moderate gonadotoxicity group, which although included patients exposed to alkylating agent chemotherapy below CED 7 grams/m^2^ and indeed any other chemotherapy, rather surprisingly also included patients who had a hysterectomy or unilateral oophorectomy. Targeted agents were also included in the “moderate” risk group, despite the near-complete absence of evidence regarding their gonadotoxicity. The authors highlight the difficulties and limitations of their classification in relation to overlapping AMH results, but this seems inevitable given that this classification was the starting point of their analysis, rather than determining a degree of reduced ovarian function, which could then be related to particular therapies.

It is noteworthy that the participants were predominantly Caucasian and well-educated. Approximately half had had either breast cancer or lymphoma, with some diagnoses, notably leukemia, being underrepresented and thyroid cancer being overrepresented. The authors used an innovative design of a combination of longitudinal and cross-sectional analysis, with a functional principal components analysis to allow for the irregular spacing and sparseness of the data. The key findings were that in all groups there was an increase in AMH levels in the initial 2 years after treatment, although the values remained low. Thereafter, there was a fairly long-lasting plateau, followed by a decline to very low levels. The high gonadotoxicity group differed somewhat from the other 2 groups, with a shorter duration of postrecovery plateau and, in general, lower AMH levels throughout. The findings were not related to a diagnosis of POI or a self-reported final menstrual period, nor were any of the details of the treatment received analyzed. These results, therefore, are largely confirmatory of previous smaller studies, with the key advances being the large number of participants, the very long time since diagnosis in some patients, and, particularly, the novel use of self-collected dried blood spots. The wide variation in the number of primordial follicles within the ovaries of women at similar ages may be a major factor in contributing to the overlapping results of the different risk groups. The absence of a pretreatment sample, which we and others have found to be an important predictor of postchemotherapy ovarian function (8), is an important limitation.

The data set that the authors have collected contains a wealth of information on treatment and subject variables, and it is likely that further analyses will provide substantially more detailed information. The use of dried blood spots also deserves wider exploration and validation in this and other contexts, in particular in relation to the developing use of AMH in the prediction of imminent menopause (9). There is still some way to go, therefore, before what the individual patient needs (ie, an accurate prediction of future reproductive function and lifespan) can be provided, but this paper provides a significant advance in the use of AMH as a valuable biomarker in the field of postcancer ovarian function and, by broader implication, in women’s long-term health after cancer.

## Additional Information

***Disclosure Summary:*** R.A.A. has undertaken consultancy work for Roche Diagnostics. W.H.B.W. has nothing to disclose.

***Data Availability:*** Data sharing is not applicable to this article as no datasets were generated or analyzed during the current study.
